# Enhanced desalination using a three-layer OTMS based superhydrophobic membrane for a membrane distillation process

**DOI:** 10.1039/c8ra01043a

**Published:** 2018-03-06

**Authors:** Saikat Sinha Ray, Shiao-Shing Chen, Hau-Ming Chang, Cao Ngoc Dan Thanh, Huy Quang Le, Nguyen Cong Nguyen

**Affiliations:** Institute of Environmental Engineering and Management, National Taipei University of Technology No. 1, Sec. 3, Zhongxiao E. Rd Taipei-10608 Taiwan f10919@ntut.edu.tw; Faculty of Environment and Natural Resources, Dalat University Vietnam

## Abstract

Superhydrophobic membranes are essential for improved seawater desalination. This study presents the successful casting of a three-layered membrane composed of a top superhydrophobic coating onto a polypropylene (PP) mat through simple sol–gel processing of octadecyltrimethoxysilane (OTMS), and the bottom layer was casted with hydrophilic poly(vinyl alcohol) (PVA) by using a knife casting technique; this membrane represents a novel class of improved-performance membranes consisting of a top superhydrophobic coating onto a hydrophobic PP mat and a hydrophilic layer (PVA) at the bottom. OTMSs are well known low-surface-energy materials that enhance superhydrophobicity, and they were observed to be the ideal chemical group for increasing the hydrophobicity of the PP mat. The PVA layer acted as base layer absorbing the condensed vapor and thus enhancing the vapor flux across the membrane. The hybrid three-layered membrane exhibited superhydrophobicity, with an average contact angle of more than 160°, and demonstrated high performance in terms of rejection and water flux. This study also examined the pore size distribution, surface roughness, surface area, tensile strength, water flux, and salt rejection of the fabricated membrane. The salt rejection level was calculated to be 99.7%, and a high permeate flux of approximately 6.7 LMH was maintained for 16 h.

## Abstract 2

Superhydrophobic membranes are essential for enhanced desalination by utilizing MD.

## Graphical abstract

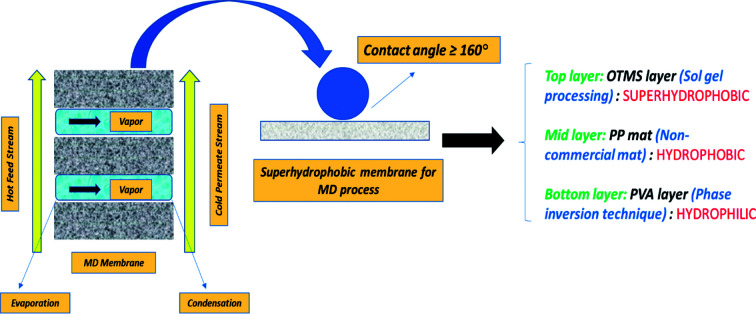

## 1. Introduction

The demand for fresh water has increased gradually in the past 20 years. Membrane distillation (MD) is one of the most effective technologies for seawater desalination. MD is a thermally driven separation process in which only vapor molecules can pass through a porous hydrophobic membrane.^1,2^ Typically, MD possesses many unique features such as requiring lower operating temperatures compared with those encountered in conventional processes, and placing lower demands on membrane mechanical strength. In addition, the hydrostatic pressure encountered in MD is much lower than that in pressure-driven membrane processes such as reverse osmosis. Hence, MD is a cost-effective process that places lower demands on membrane characteristics.^2–4^

Hydrophobic materials such as polyvinylidene fluoride (PVDF), polypropylene (PP), and polytetrafluoroethylene (PTFE) are generally utilized in the MD process, and they are fabricated through processes such as stretching, electrospinning, thermally induced phase separation, and phase inversion.^5,6^ Direct contact membrane distillation (DCMD) configuration is a type of MD in which an aqueous solution at a lower temperature is in direct contact with the permeate stream of the membrane. DCMD configuration has been widely studied because of its convenience and simplicity.^7^

To prevent membrane wetting and the subsequent formation of liquid-filled pores with higher mass transfer resistance, hydrophobic membranes composed of PVDF and PTFE are commonly utilized in MD.^8^ Commercial membranes such as PP, PTFE, and PVDF demonstrate higher hydrophobicity and chemical and thermal resistance, compared with other membranes. Recently, membrane researchers have further modified these MD membranes into superhydrophobic membranes to avoid membrane fouling by reducing the direct contact between membrane foulants and membrane surface at the Cassie–Baxter state.^9^ Superhydrophobic-specialized surfaces with versatile features have gained increased attention in the field of desalination and filtration. In general, MD processes must generate pure water with lower conductivity continuously. However, liquid penetration and pore wetting during long-term operation lead to lower salt rejection. According to some researchers, an increase in the hydrophobicity of MD membranes can reduce membrane pore wetting effectively.^10,11^ Therefore, researchers are attempting to develop superhydrophobic membranes by utilizing hydrophobic additives.^12^ Superhydrophobicity introduces an air gap between water molecules as well as on the membrane surface, which can potentially increase the allowable pore sizes before pore wetting occurs, resulting in a higher mass flux.^13^ In addition, superhydrophobic modification could reduce heat loss through conduction across the membrane^14^.

**Fig. 1:**
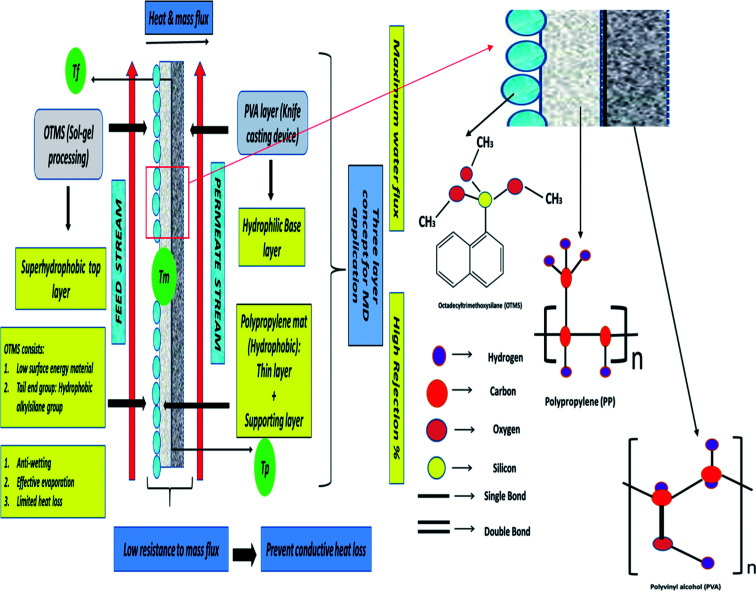
Schematic illustration of three layered membrane for MD application.

In this study, a three-layered membrane was fabricated for enhanced desalination in MD applications. Octadecyltrimethoxysilane (OTMS) is well known as a low-surface-energy material. OTMS have 18 carbon atom hydrocarbon chains and tail end group of OTMS molecule is the hydrophobic alkylsilane group, and the monolayers of OTMS self-assemble on a solid surface, with their molecular axis perpendicular to the surface.^15^ Typically, OTMS layers are applied to lower the surface energy of solid surfaces to render superhydrophobic characteristics. OTMS materials are utilized as one of the coprecursors in sol–gel processing to achieve superhydrophobic coatings or layers.^16,17^ In this study, a simple and single-step sol–gel processing of long-chain OTMS was performed to fabricate a superhydrophobic layer on a PP mat. The fabricated coatings were transparent, were chemically durable, and exhibited an attractive self-cleaning property. The OTMS coating was the top-most layer and was determined to be superhydrophobic, the PP mat was the middle layer and was observed to be hydrophobic as the contact angle was found to be 99.5° and in addition to that the pore size and thickness was evaluated to be 20 nm and 25 μm respectively. Poly(vinyl alcohol) (PVA) was the bottom layer and was determined to be hydrophilic; the PVA layer was casted by using a knife casting technique. Here, as mentioned earlier OTMS coating was the top superhydrophobic layer as the contact angle was determined to be more than 160°, the PP mat acted as a porous supportive layer improving the mechanical stability during the long-term MD operation. Additionally, the PVA layer could absorb condensed vapor, thus directly enhancing the vapor flux across the membrane.^18^ The hydrophilic bottom layer could facilitate the absorption of the condensed vapor from the superhydrophobic top layer, thus preventing pore wetting. Moreover, the combination of the superhydrophobic/hydrophilic layer could provide low resistance to mass flux and also prevent conductive heat loss. Therefore, the three-layered OTMS-PP/PVA contributed to improved mass flux. Fig. 1 demonstrates the detailed mechanism of the three-layered surface membrane composed of OTMS-PP/PVA. The figure also indicates the reasons for placing the superhydrophobic OTMS-PP layer on the feed solution side.

Recently in market, microporous hydrophobic membranes made of PP, PTFE, and PVDF are commercially available. Typically, PTFE is the most ideal polymer for MD membrane because of its hydrophobic characteristic, good chemical resistance and thermal stability. Various commercially available membranes used in MD process has been summarised in Table 1. The general properties of the proposed membrane have been compared with that of other commercially available membranes based on membrane thickness, average pore size, contact angle. The present composite membrane achieves comparable membrane thickness and average pore size which makes it feasible for direct application in MD system.

**Table 1:** Comparison between commercial and casted membrane for MD process

Material	Manufacturer	Conditions	Salt rejection%	Membrane thickness, (μm)	Average pore size, (μm)	Contact angle, (°)	References
PVDF/nonwoven	GE Osmonics	*T*_f_ = 80 °C, *T*_p_ = 20 °C	∼99%	234	0.30	113	19
PVDF	PV390, Memteck	*T*_f_ = 70 °C, *T*_p_ = 20 °C	∼99%	115	0.26	97	20
PTFE	Membrane solution	*T*_f_ = 80 °C, *T*_p_ = 20 °C	∼99.2%	70	1.00	130	21
PVDF	Millipore	*T*_f_ = 80 °C, *T*_p_ = 20 °C	∼99.1%	118	0.27	110	22
OTMS-PP/PVA	—	*T*_f_ = 70 °C, *T*_p_ = 20 °C	∼99.8%	100	0.06–0.1	162	Present study

The fabrication of the composite membrane is simple and includes only three steps: (a) deposition of PP mat in OTMS solution and dried for 12 h at room temperature; (b) PVA solution was used to fabricate a hydrophilic layer by utilizing knife casting device and (c) the composite membrane OTMS-PP/PVA undergone heat-pressing treatment. An elaborate discussion has been provided in Section 2.2.

Current research has focused on improving membranes by modifying the surface chemistry.^23,24^ The current study examined factors such as high permeate water flux and higher salt rejection (≥99%); therefore, it also confirmed the feasibility of the proposed three-layered OTMS-PP/PVA membranes for MD applications. The overall MD performance of the fabricated membrane was carefully analyzed using a thermally driven MD process to calculate the permeate water flux and salt rejection of 30 000 ppm NaCl aqueous solution. Furthermore, this paper reports the fundamental concepts for casting other novel three and bilayered superhydrophobic membranes for MD applications.

## 2. Materials and methods

### 2.1 Starting material

OTMS with a molecular weight of 586.30 was ordered from Matrix Scientific. PVA with a molecular weight of *M*_n_ 146 000–186 000 and 99.9% hydrolyzed was purchased from Sigma-Aldrich. Furthermore, PP mats (non-commercial) were provided by the BenQ Material Corporation, Taiwan.

### 2.2 Experimental

#### 2.2.1 Preparation of superhydrophobic material

First, 1 mL of distilled water and 0.5 mL of ammonia were mixed thoroughly with 25 mL of ethanol and stirred for 10 min in a magnetic stirrer. Subsequently, 200 μL of OTMS was slowly added to this solution under constant stirring. Ethanol was utilized as a solvent, whereas ammonia was applied as a catalyst to initiate the sol–gel process, However, water was used to accelerate the hydrolysis reaction. Typically, a functional methoxy group of OTMS undergoes hydrolysis and polycondensation reactions to form network-like structures. Finally, after 10 min, a homogenous solution appeared, confirming the network structure formation in the sol.

#### 2.2.2 Preparation of superhydrophobic coating by sol–gel process

First, the PP mats were cleaned thoroughly and then immersed in the aforementioned solution for deposition times of 5, 15, and 30 min. The superhydrophobic coatings prepared with deposition times of 5, 15, and 30 min were named as OTMS(5), OTMS(15), and OTMS(30), respectively. A thin, watery coating was formed on the precleaned PP mats. Finally, the superhydrophobic coatings were dried for 12 h at room temperature.

#### 2.2.3 Preparation of PVA solution for knife casting

PVA was dissolved in distilled water of 8% w/v concentration inside a water bath maintaining a temperature of up to 120–140 °C under constant stirring of 700–800 rpm in a magnetic stirrer. A thick solution was obtained after maintaining the same condition for 10–12 h. Because the molecular weight of PVA is high, dissolving PVA polymers higher than 8% w/v is very difficult.^25^ The controlled conditions for the membrane fabrication are mentioned in Table 2.

**Table 2:** Controlled conditions for fabrication of PVA layer by using knife casting device

Polymer concentration	Casting temperature	Relative air humidity	Casting vacuum plate speed	Casting knife gap
8% w/v PVA	24 ± 2 °C	31 ± 5%	10 mm s^−1^	100 μm

#### 2.2.4 Membrane distillation application

**Fig. 2:**
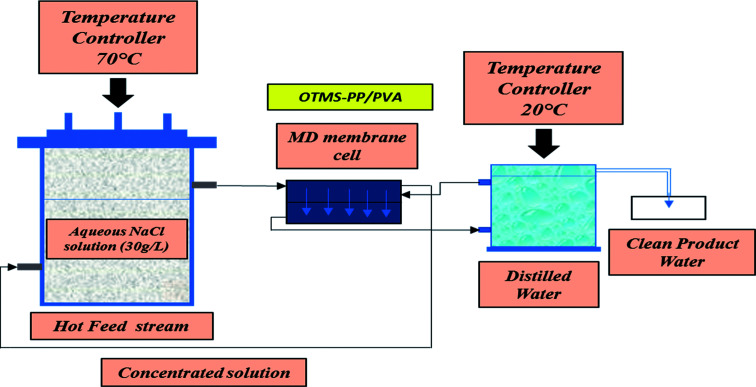
Schematic of lab-scale direct contact membrane distillation system.

MD was performed at lab scale as indicated in Fig. 2. The MD experimental test cell lab-scale setup was acquired from Sterlitech Corporation Limited. The observations were carefully analyzed for different durations from 1 until 16 h, and the results were evaluated for different temperature differences. Fig. 2 presents the schematic lab setup of the MD process.

The water flux *J*_w_ (L m^−2^ h^−1^; LMH) was evaluated in terms of the total volume of the permeate water by using eqn (1)^2,26–28^1
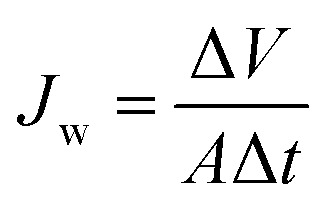
where *V* is the total volume of the permeate over a specific time period Δ*t* (h) and *A* is the effective surface area of the membrane utilized in the MD process. The salt rejection of the system, indicated as the percentage of NaCl retained by the membrane, was measured by using eqn (2).^26^2
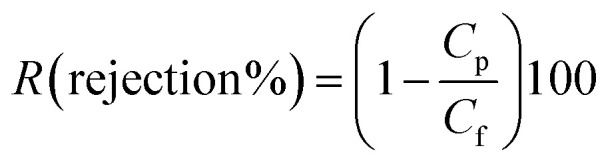
where *C*_P_ (g L^−1^) is the solute concentration in the permeate stream side and *C*_f_ (g L^−1^) is the solute concentration on the feed stream side.

### 2.3 Characterization

The surface and cross-sectional morphologies were thoroughly examined through scanning electron microscopy (SEM; JEOL JSM-5900, Japan). The average contact angle of the membrane was studied using an optical surface analyzer (OSA60-G, Ningbo NB Scientific Instruments Co., Ltd., China) to measure its hydrophobicity and hydrophilicity. The surface roughness was analyzed through atomic force microscopy (AFM). Membrane characteristics such as pore diameter, pore width, surface area, and pore volume were examined using an adsorption/desorption analyzer (Micromeritics N_2_, ASAP 2020, USA).

### 2.4 Quality control

To obtain reliable data, the experiments were executed in triplicate and the mean values were recorded. Error bars are based on the standard errors of the three replicate results.

## 3. Results and discussions

### 3.1 Characterization of the membranes

#### 3.1.1 Average contact angle evaluation

**Fig. 3:**
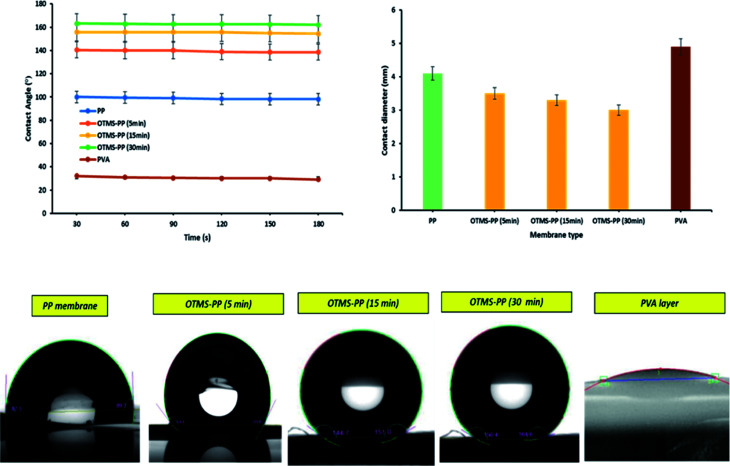
Analysis of average contact angle and contact diameter of various membranes [note: error bars are based on standard errors from three replicate tests].

When an interface occurs in between liquid and solid, the angle between the liquid surface and the outline of the contact surface is indicated as the contact angle. Generally, contact angle is the measure of wettability of a solid by liquid. In complete wetting, the contact angle tends to 0°, whereas, the solid is wettable in between 0° and 90°, and above 90° it is considered to be non-wettable. Thus, the materials with higher contact angle (superhydrophobic) are supposed to have anti-wetting property.^29^ The average contact angle of the PP mat and modified PP membranes (OTMS-PP/PVA) were carefully analyzed using an optical surface analyzer (OSA60-G, Ningbo NB Scientific Instruments Co., Ltd., China). Typically, the average contact angle is considered to illustrate the nature of the membrane surface in terms of hydrophobicity or hydrophilicity. In this study, the average contact angle of the PP mat was observed to be hydrophobic. After the surface chemistry modification of the PP mat with OTMS, the average contact angle demonstrated a superhydrophobic nature. The contact angle of the modified surface of the PP mat increased as the deposition time of OTMS increased from 5 to 30 min through sol–gel processing. Finally, the bottom layer was cast through a knife casting device by using PVA, which was observed to be highly hydrophilic. Fig. 3 indicates the average contact angle analysis of the membrane surfaces used in the MD application. The contact diameter was measured, and the data suggested that a higher average contact angle was correlated with a lower average contact diameter. Fig. 3 indicates that OTMS (30 min)-PP had the highest average contact angle (163°) and the lowest contact diameter (3.0 mm).

#### 3.1.2 SEM morphology analysis

**Fig. 4:**
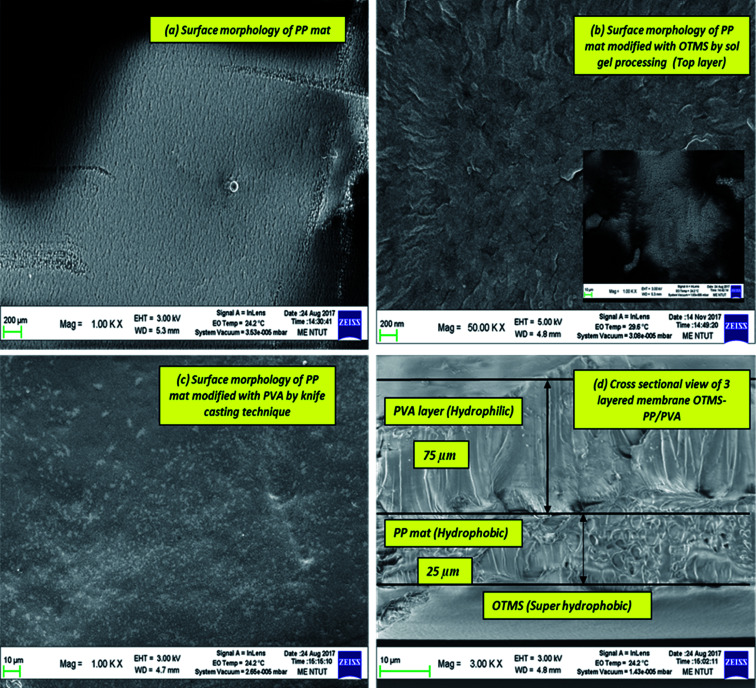
SEM micrographs analysis: (a) surface morphology of polypropylene (PP) mat (middle layer); (b) morphology of PP mat modified with OTMS chemical group (top layer); (c) morphology of PP mat modified with PVA layer (bottom layer); (d) cross sectional view of three layered membrane OTMS-PP/PVA.

The uniformity and morphology of the coatings were thoroughly characterised by SEM and SEM micrographs are indicated in Fig. 4. The morphology of the fabricated membranes was examined by using field emission SEM (JOEL, JSM 7600 F, Japan). The OTMS layer must be placed on top to avoid them being covered by the PP mat. Fig. 4 indicates the morphological study results, presenting multiple SEM images to illustrate the effect of surface chemistry after sol–gel processing of the PP mat through OTMS. There is no such change in the morphology of PP mat modified with OTMS as the coating of OTMS tends to be very thin self-assembled monolayers. Thus, it can be seen that the morphology appears to be quite similar before and after sol gel treatment technique. Additionally, the bottom PVA layer was analyzed through SEM. The cross-sectional view of the membrane is also added to this figure. This figure clearly indicates the successful fabrication of the OTMS-PP/PVA membrane.

#### 3.1.3 Surface roughness analysis by AFM

**Fig. 5:**
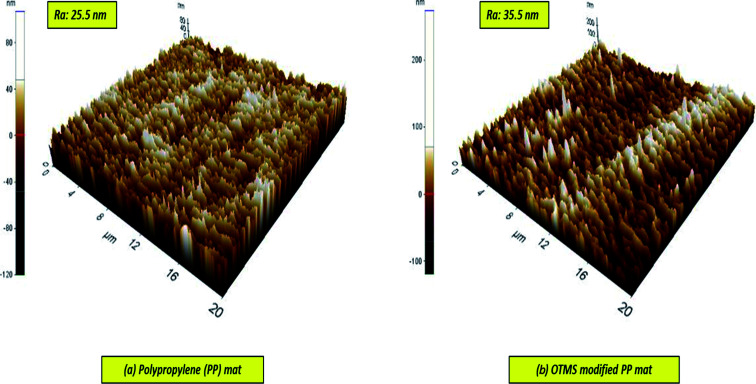
AFM 3-D micrographs of membrane surfaces before and after modification by OTMS chemical group (dimensions: 20 μm × 20 μm).

Typically, lower surface energy and surface roughness play an important role in the wetting features of solid surfaces. The surface morphologies of the coatings of the modified membranes are depicted in Fig. 5. A wrinkled, rough, mountain-like nanostructure appeared before and after the coating of the PP mat with OTMS. The figure indicates that the surface roughness doesn't show significant change and increased a bit from 25.5 to 35 nm. The OTMS (30 min)-PP/PVA membrane had a maximum contact angle of 163°. Furthermore, the surface roughness lowers the total area of solid–liquid interface and increases the air pockets on the membrane surface, which makes the water droplets move more easily and hence, reduces the water sliding angles. Typically, lower the sliding angle the easier to clean the surface.^30^

#### 3.1.4 Membrane surface analysis through Barrett–Joyner–Halenda adsorption/desorption technique

**Fig. 6:**
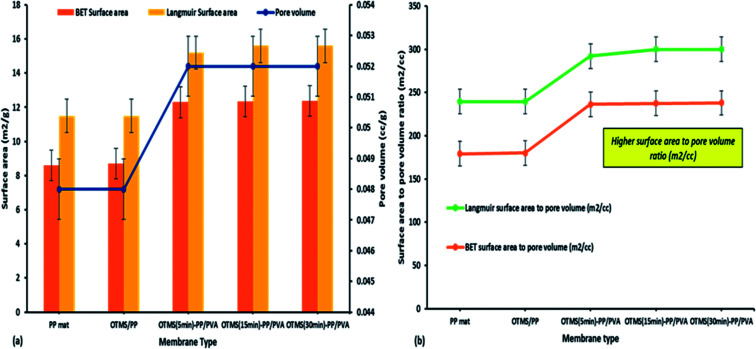
Surface area and pore volume analysis: (a) BET and Langmuir surface area with pore volume analysis; (b) surface area to pore volume ratio of different fabricated membranes [note: error bars are based on standard errors from three replicate tests].

The pore size distribution provides quantitative information on the pore size ranges present in a membrane sample. Typically, it indicates a more specific description of the particle sizes likely to be removed or retained by the membrane.^31–33^ The membrane surface was examined using a Micromeritics N_2_ adsorption/desorption analyzer (ASAP 2020, USA). The data included the Langmuir surface area, Brunauer–Emmett–Teller (BET) surface area, pore volume, pore width, and pore diameter. The Langmuir and BET surface areas appeared to increase with the deposition time of OTMS onto PP/PVA; very little differences in the pore volume were observed. Fig. 6 presents the surface area-to-pore volume ratio. Notably, because of the high surface area-to-pore volume ratio, these novel types of fabricated MD membranes could overcome the low flux limitation of the PP mat. This effect could be observed in the permeate water flux, which is discussed in the next section.

The membrane thickness of the PP mat was measured to be 20 μm, which is very thin compared with other commercial MD membranes. Subsequently, the thickness of the fabricated membrane was observed to increase by four times. Typically, the membrane thickness has an effect on water flux and reduce the thermal resistance by lowering the heat efficiency or interface temperature difference as the membrane becomes thinner. Additionally, higher surface area to pore volume ratio plays an important role in lowering the mass transfer resistance.^34^ This effect can be observed in permeate water flux, which has been discussed in next section.

**Fig. 7:**
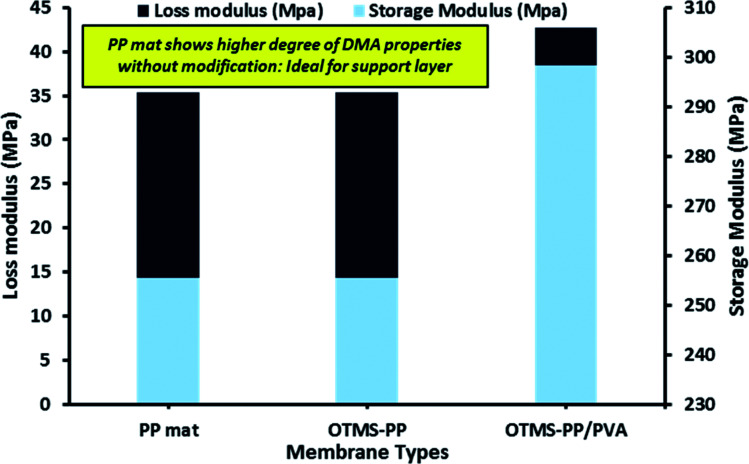
Dynamic mechanical analysis (DMA) of different fabricated membrane in terms of loss modulus and storage modulus.

Fig. 7 indicates the dynamic mechanical analysis of different membranes in terms of storage modulus and loss modulus. The loss modulus is the measure of energy dissipation, though as a modulus, the mechanical strength of a material is defined by its hardness or stiffness.^35^ The thickness of the surface-modified MD membranes was determined to be comparable to those of commercially available membranes for MD processes (range: 80–170 μm).

Loss modulus is the basic property to characterise the viscoelastic performance of a material. Moreover, higher storage modulus denotes more solid-like property and in simple words, higher the storage modulus indicates higher strength or mechanical rigidity. Infact, this is directly co-related to the degree of material integrity. Thus, higher the degree of material integrity the greater the storage modulus.^36^

**Fig. 8:**
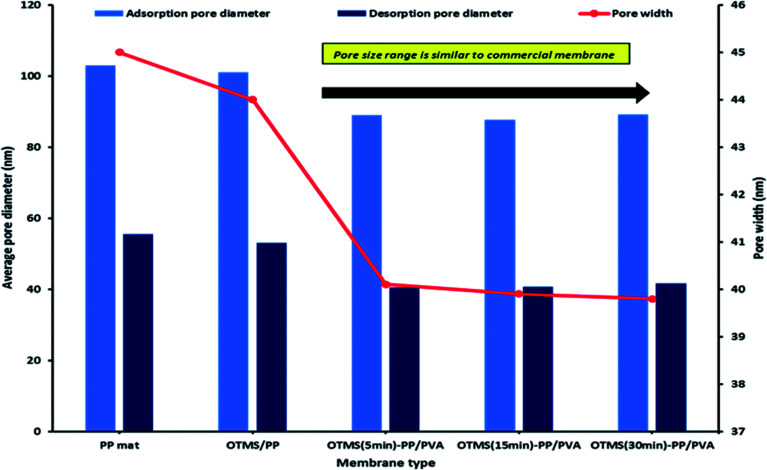
Barrett–Joyner–Halenda (BJH) analysis of adsorption/desorption average pore diameter and pore width for different modified membranes.

In this study, we analyzed both the pore width and pore diameter. The pore width and diameters of the PP mat and modified OTMS-PP/PVA membranes were examined using Barrett–Joyner–Halenda adsorption/desorption techniques. The differences observed in pore diameter were not significant. However, the OTMS-PP/PVA membrane was expected to exhibit a higher salt rejection than the PP mat due to its lower pore diameter range. To support the previous results, the average pore widths of these fabricated membranes were also examined, and OTMS-PP/PVA was observed to possess a very small pore width of 30 nm compared with that of the PP mat. Fig. 8 denotes the average pore width and average pore diameter of all the fabricated membranes including the commercial PP mat. Interestingly, the adsorption/desorption average pore diameters and pore widths were found to be similar for different OTMS-PP/PVA membranes as the composition and fabrication technique of all these membranes are similar. Though the superhydrophobic OTMS coating prepared with deposition times of 5, 15, and 30 min were observed to be insignificant in influencing the pore diameter and pore width.

Membrane wetting can be examined by average contact angle. Typically, lower pore size, higher contact angle and surface tension increase liquid entry pressure (LEP). As earlier mentioned, membrane wetting pores leads to reduced permeate quality; hence, it is necessary to utilize membranes with greater LEP value.^37^ Franken *et al.*^38^ has suggested a model to evaluate LEP value based on Cantor–Laplace equation^38^eqn (3):3
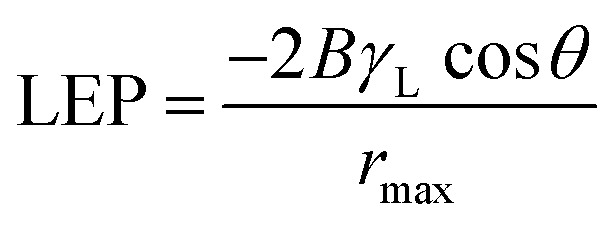
where LEP represents liquid entry pressure of pure water in Pa, *B* represents dimensionless geometrical factor which includes the irregularities of the pores (*B* = 1 for assumed cylindrical pores), *γ*_L_ represents the liquid surface tension in N m^−1^ (in this case water at 25 °C, 0.07199 N m^−1^), cos *θ* represents the contact angle in degree, *r*_max_ is the maximal pore (non-closed) radius in m.

**Fig. 9:**
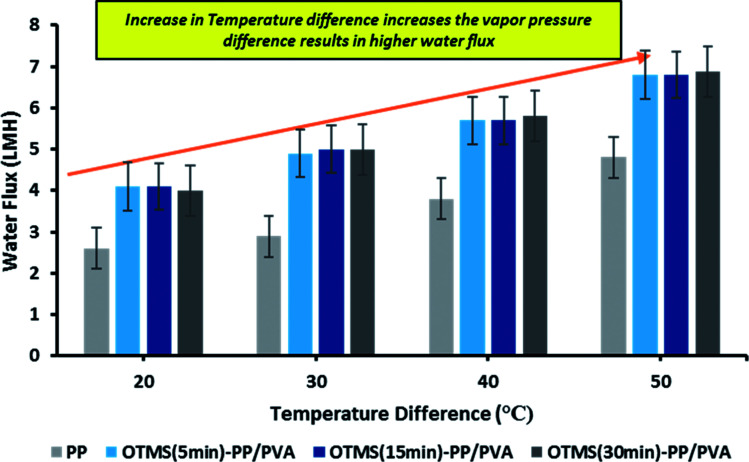
Effect of temperature difference on permeate water flux in membrane distillation [note: feed solution = 30 g L^−1^ NaCl solution, time period = 1 h]. [Note: error bars are based on standard errors from three replicate tests].

**Fig. 10:**
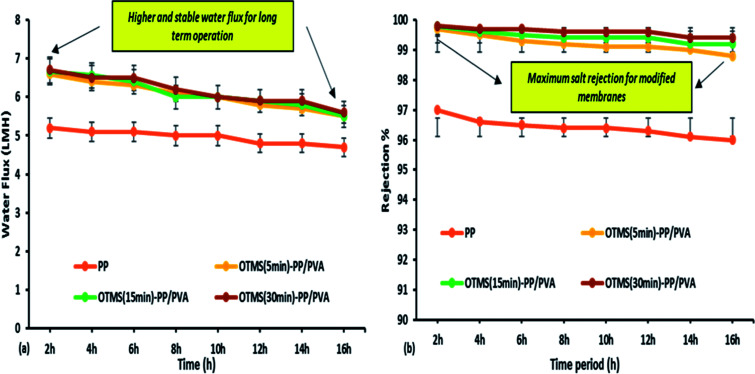
MD performance of various membranes (a) effect of time interval on water flux; (b) effect of time interval on salt rejection% of fabricated membranes used in the MD process [note: feed solution = 30 g L^−1^ aqueous NaCl solution, temperature difference = 50 °C]. Error bars are based on standard errors from three replicate tests.

Table 3 represents the LEP values that was calculated using the Cantor–Laplace equation. Interestingly, the fabricated membranes OTMS-PP/PVA show higher degree of LEP than compared to PP mat which indirectly indicates reduced wetting probability. This effect can be clearly seen in Fig. 9 and 10.

**Table 3:** Summarisation of membrane properties based on LEP [note: LEP was calculated by using Cantor–Laplace equation; 1 Pa = 10^−5^ bar]

Membrane type	Average contact angle, (°)	LEP, (bar)
PP mat	99.5°	0.30
OTMS (5 min)-PP/PVA	140.1°	4.44
OTMS (15 min)-PP/PVA	158.7°	4.75
OTMS (30 min)-PP/PVA	163°	4.96

### 3.2 Membrane performance in membrane distillation

Lab-scale MD was performed as shown in Fig. 2. NaCl solution with a concentration of 30 000 ppm was used as the feed stream solution. The temperature of the feed stream was maintained using a water bath and was varied between 40 and 70 °C. By contrast, the temperature of the distillate (cooling) stream was controlled at 20 °C by circulating a large quantity of distilled water through an air-cooling system. The membrane area was evaluated to be 100 cm^2^ (dimensions: 10 cm × 10 cm). The salt concentration of the feed stream and the permeate water quality were analyzed by a professional series and a conductivity meter/TDS/DO (YSI Quatro, USA). The overall outcomes of the MD process indicated a steep increase in the permeate water flux with an increasing temperature difference up to 70 °C (Δ*T* = 50 °C). The permeate water flux varied from 3.5 to 6.7 LMH, indicating a stronger temperature influence. OTMS-PP/PVA demonstrated the highest permeate water flux, compared with the PP mat. Fig. 9 reveals an increase in the permeate water flux with an increase in temperature difference for the different fabricated membranes used in the MD process.

The MD process was continued for up to 16 h for the different fabricated membranes to examine their long-term stability performance. However, the permeate water flux and salt rejection decreased slightly as the time increased. OTMS (30 min)-PP/PVA demonstrated the highest water flux of 6.7 LMH and salt rejection of 99.89%. From Fig. 10, we can conclude that the negligible decline in water flux over time clearly demonstrates good stability and durability of the OTMS and PVA sublayers on the PP mat. Specifically, the interconnectivity and stability of these two sub layers (top layer and bottom layer) on the PP mat can be demonstrated by the stable water flux and rejection until 16 h of operation. Furthermore, the salt rejection of all the fabricated membranes was found to be 99.3–99.89%, illustrating good permeating quality on the permeate side as compared with the PP mat, which can be clearly seen in Fig. 10.

**Fig. 11:**
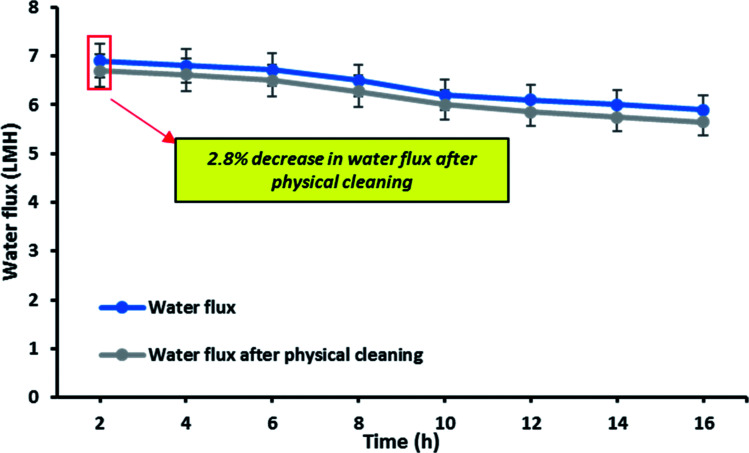
Analysis of reusability of membrane in terms of water flux decline: graphical representation of water flux and water flux after physical cleaning [note: membrane used: OTMS-PP/PVA, time: 16 h, feed stream: 30 g L^−1^, *T*_f_: 70 °C, *T*_p_: 20 °C].

Fig. 11 shows the reusability of the OTMS-PP/PVA membrane which was examined by evaluating the decrease in water flux after physical cleaning of the membrane. In this experiment, the same membrane was reused for 16 h to analyse the long-term stability as well as reusability of the membrane. Interestingly, only 2.8% decrease in water flux was evaluated. Therefore, it can be concluded that the reduction in water flux seems to be insignificant after reusing it for 16 h which directly denotes the self-cleaning property of this OTMS-PP/PVA superhydrophobic membrane.

## 4. Conclusion

In this study, a three-layered membrane consisting of a top superhydrophobic coating was cast onto a PP mat through a simple one-step sol–gel processing of long-chain OTMS, and a base layer of PVA was fabricated through a knife casting technique, which has been successfully utilized for MD desalination. The PVA layer acted as a bottom layer absorbing water molecule (condensed vapor) and thus enhancing the vapor flux across the membrane. The results demonstrate that the hybrid membrane composed of a superhydrophobic coating of OTMS onto a PP mat exhibited superhydrophobicity, with an average contact angle of approximately 163°. Additionally, the membrane's morphology, average contact angle, pore size, surface area, and surface roughness were thoroughly examined and compared with those of a standard PP mat. The water flux and salt rejection percentage were also analyzed using 30 g L^−1^ of NaCl solution in the feed stream. Changes in the surface chemistry of the modified three-layered OTMS-PP/PVA membranes resulted in a higher pore volume, producing a higher permeate flux when compared with the PP mat. Furthermore, this three-layered membrane achieved a salt rejection level of 99.7%. In conclusion, the OTMS-PP/PVA membrane achieved an improved salt rejection and a stable permeate flux, confirming the feasibility of a three-layered membrane for long-term MD operations.

## Conflicts of interest

There are no conflicts to declare.

## Supplementary Material
